# GONAD: A new method for germline genome editing in mice and rats

**DOI:** 10.1111/dgd.12746

**Published:** 2021-10-05

**Authors:** Masumi Namba, Tomoe Kobayashi, Takayuki Koyano, Mayumi Kohno, Masato Ohtsuka, Makoto Matsuyama

**Affiliations:** ^1^ Division of Molecular Genetics Shigei Medical Research Institute Okayama Japan; ^2^ Department of Molecular Life Science Division of Basic Medical Science and Molecular Medicine Tokai University School of Medicine Isehara Japan; ^3^ The Institute of Medical Sciences Tokai University Isehara Japan

**Keywords:** CRISPR/Cas9, *i*‐GONAD, in vivo electroporation, knock‐out, rGONAD

## Abstract

Recent advances in the CRISPR/Cas9 system have demonstrated it to be an efficient gene‐editing technology for various organisms. Laboratory mice and rats are widely used as common models of human diseases; however, the current standard method to create genome‐engineered animals is laborious and involves three major steps: isolation of zygotes from females, ex vivo micromanipulation of zygotes, and implantation into pseudopregnant females. To circumvent this, we recently developed a novel method named Genome‐editing via Oviductal Nucleic Acids Delivery (GONAD). This method does not require the ex vivo handling of embryos; instead, it can execute gene editing with just one step, via the delivery of a genome‐editing mixture into embryos in the oviduct, by electroporation. Here, we present a further improvement of GONAD that is easily applicable to both mice and rats. It is a rapid, low‐cost, and ethical approach fulfilling the 3R principles of animal experimentation: Reduction, Replacement, and Refinement. This method has been reconstructed and renamed as “improved GONAD (*i*‐GONAD)” for mice, and “rat improved GONAD (rGONAD)” for rats.

## Graphical abstract

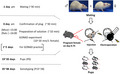

**Highlights**

- Intended organisms: Mice and rats
- Purpose of the protocol: Production of genome‐edited mice and rats
- Essential equipment: Electroporator, mechanical pipette puller
- Features: This method does not require (1) professional skills such as embryo manipulation and microinjection, nor (2) the sacrificing of pregnant mice and rats to isolate zygotes; therefore, it is simpler, easier, and quicker than conventional protocols using microinjection and uterine foster mothers.

## 1 BACKGROUND AND FEATURES

The recently developed CRISPR/Cas9 system is the most convenient and reliable method for generating animals carrying modified genomes. This system efficiently generates animals with the need for “knock‐out” and “knock‐in” of target sequences (Atanur et al., 2013; Gurumurthy, Grati, et al., 2016). However, for most laboratories, the genome engineering of mammals is a difficult and laborious task because the most common procedures for creating genome‐edited mammals include three major ex vivo embryo handling steps (Li et al., 2013), namely, (1) the isolation of zygotes, (2) the microinjection of zygotes, and (3) the transfer of microinjected zygotes into the oviducts of pseudopregnant females. These three steps require the researchers and technicians performing the procedures to be proficient in highly technical skills, and necessitate the use of expensive apparatus such as micromanipulators.

To simplify these complex and laborious processes, we established a novel genome engineering method, named Genome‐editing via Oviductal Nucleic Acids Delivery (GONAD), for mice (Gurumurthy, Takahashi, et al., 2016; Gurumurthy et al., 2019; Ohtsuka & Sato, 2019; Ohtsuka et al., 2018; Takahashi et al., 2015). In this method, in vivo genome editing of early preimplantation embryos present in the oviducts of pregnant females is performed. Therefore, GONAD does not require the aforementioned ex vivo handling of embryos. In the first GONAD trial with Cas9 mRNA and sgRNA, genome‐editing efficiency was approximately 25% (Takahashi et al., 2015). In 2018, we developed a novel method for mice (*i*‐GONAD), and its efficiency was substantially improved with the use of Cas9 protein and crRNA/tracrRNA complex (knock‐out, 50–100%; knock‐in, 15–40%; Ohtsuka et al., 2018). Moreover, we established a method to produce knock‐out and knock‐in rats with high efficiency, named rat improved GONAD (rGONAD) (Kobayashi et al., 2018).

In studies involving animals, it is essential to meet the 3R principles of animal experimentation: Reduction, Replacement, and Refinement. Unlike traditional approaches, GONAD does not require the sacrifice of pregnant females to isolate zygotes; many females that undergo GONAD can deliver pups. Moreover, GONAD does not require the preparation of pseudopregnant females (obtained by mating females with vasectomized males); therefore, we can reduce the number of animals used. For these reasons, the production of genome‐edited animals with GONAD meets the 3R principles.

**FIGURE 1:**
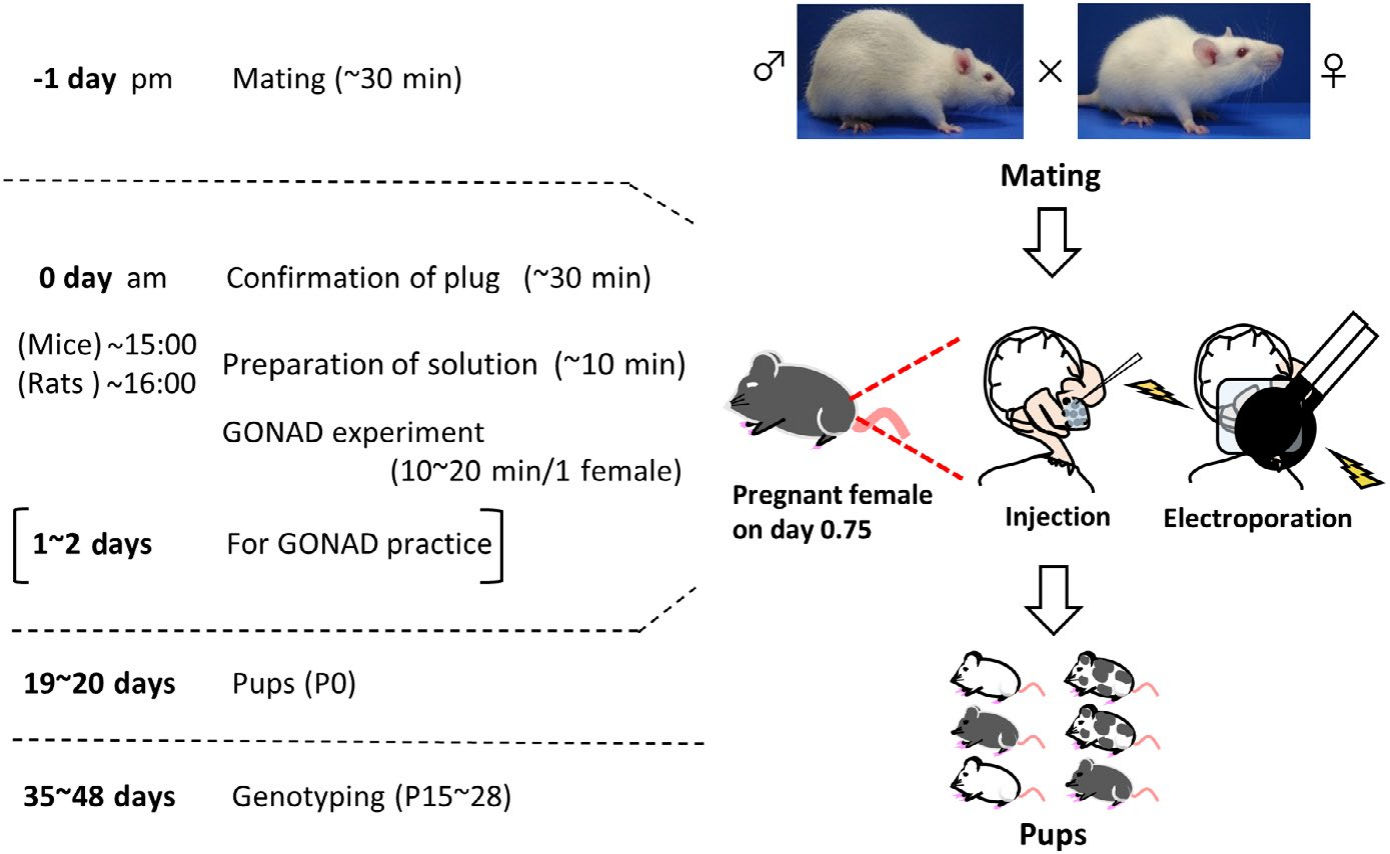
Overview of the GONAD workflow

Here, we provide a step‐by‐step protocol for GONAD with some examples of genome‐edited mice and rats. We detail rGONAD and electroporation conditions and discuss the possibility of more efficient production with biallelic disruptions than with the previous protocol (Gurumurthy et al., 2019). GONAD is simple and rapid (Figure 1), and we strongly believe that it will facilitate the production of genome‐engineered rodents in any laboratory. Furthermore, GONAD can be readily applied in mammals such as guinea pigs, hamsters (Hirose et al., 2020), cows, and pigs, in which traditional gene targeting approaches using embryonic stem cells or ex vivo embryo culture methods are not well established. Nonetheless, it is still difficult to use GONAD to introduce larger mutations requiring a long DNA donor (>2 kb). If even larger knock‐ins need to be engineered, other methods such as microinjection and CRISPR‐READI may be more suitable (Chen et al., 2019; Chenouard et al., 2021).

## 2 MATERIALS AND EQUIPMENT

### 2.1 Equipment (Figure 2)

Electroporator (e.g., NEPA21, NepaGene)
Tweezer‐type electrodes (e.g., mouse, CUY652P2.5x4; rat, CUY652P3x4.5; NepaGene)
Dissecting microscope (e.g., SZX7; Olympus)
Mechanical pipette puller (e.g., Puller PN‐31, Narishige)
Fluorescence inverted microscope (for practice) (e.g., IX73; Olympus)

**FIGURE 2:**
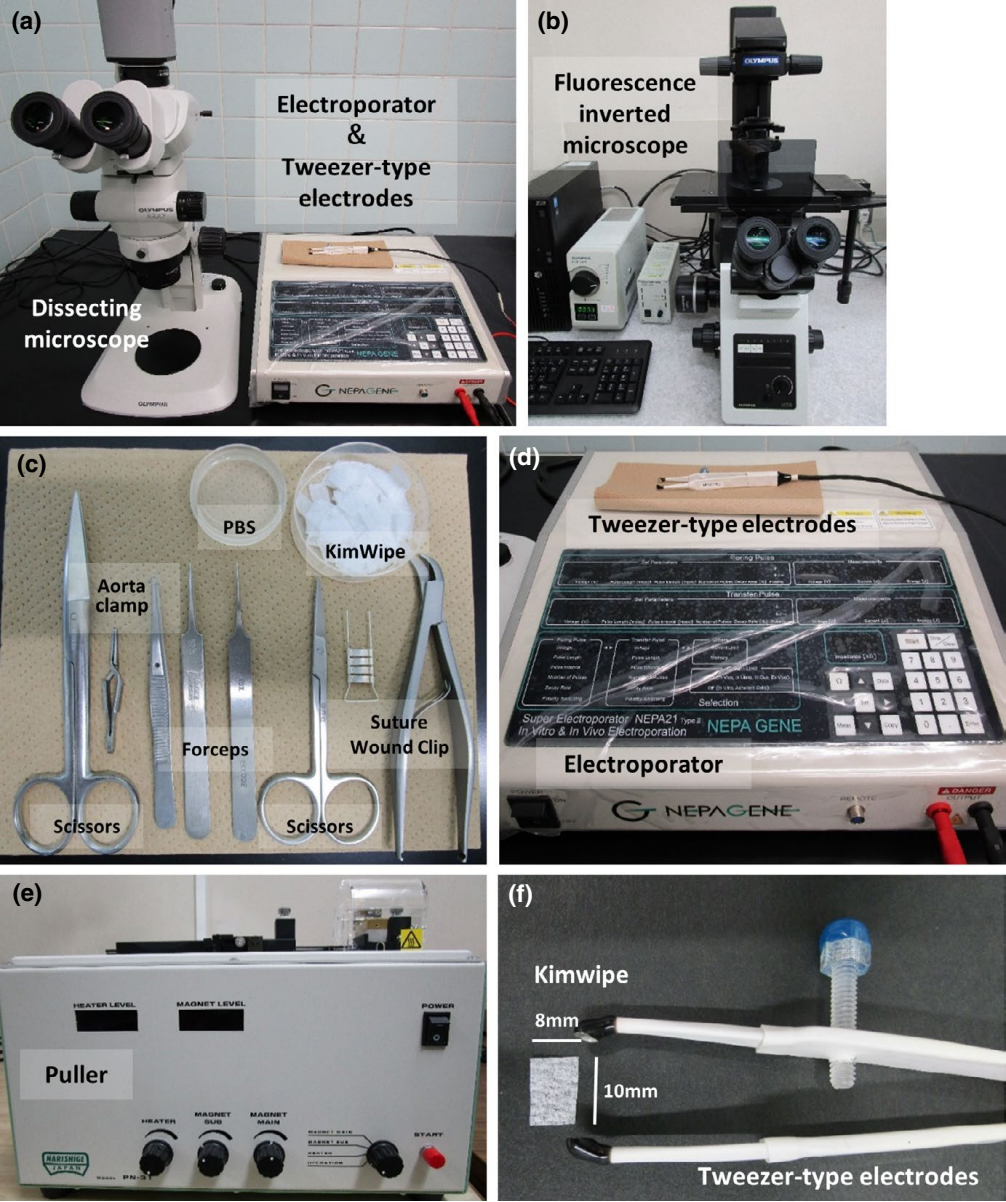
Equipment and surgical instruments. (a) SZX7 microscope and NAPA21 electroporator. (b) IX73 fluorescence inverted microscope. (c) Surgical instruments needed for GONAD. (d) NAPA21 electroporator and tweezer‐type electrodes. (e) PN‐31 puller. (f) Size of the cut KimWipe for electroporation

### 2.2 Surgical instruments (Figure 2c)

Scissors
Forceps
Aorta clamp (e.g., small tubing clamp, SANSHO, #91‐3102)
Suture wound clamp (e.g., Natsume, #C‐21‐2)
Suture wound clip (e.g., Natsume, #C‐21‐S)
Glass capillary (Drummond, #1‐000‐0300)
Aspirator tube assembly (Drummond, #2‐040‐000, or #2‐000‐000)
Paper towels (e.g., KimWipe, Crecia)

### 2.3 Reagents

crRNA (Integrated DNA Technologies: IDT, custom)
tracrRNA (IDT, 1072534)
Cas9 protein (IDT, 1081059)
Duplex buffer (IDT, 11050112)
Single‐strand DNA (ssDNA; custom, e.g., Eurofins Genomics)
1× PBS (phosphate‐buffered saline)
Opti‐MEM (Thermo Fisher Scientific, 31985062)

#### 2.3.1 Anesthetic agent

Midazolam (10 mg/2 ml; SANDOZ)
Vetorphale (5.0 mg/ml; Meiji)
Domitor (1.0 mg/ml; ZENOAQ)
0.6% NaCl

#### 2.3.2 For GONAD practice (optional)

Tetramethylrhodamine‐labeled dextran (3 kDa) (Thermo Fisher Scientific, #D3307)
Trypan blue (e.g., Nacalai Tesque, 29853‐34)
10% fetal bovine serum (FBS)/PBS

### 2.4 Mice and rats

Mice: CD‐1 (alias ICR; female, 8–20 weeks; male, 8–30 weeks), C57BL/6 (female, 8–16 weeks; male, 10–24 weeks)
Rats: WKY (female, 10–16 weeks; male, 12–24 weeks), DA (female, 10–16 weeks; male, 10–24 weeks), F344 (female, 10–16 weeks; male, 12–24 weeks), *SD* (female, 10–16 weeks; male, 10–24 weeks)

### 2.5 Equipment/reagent setup

#### 2.5.1 Micropipette (Figures 2e and 3a)

**FIGURE 3:**
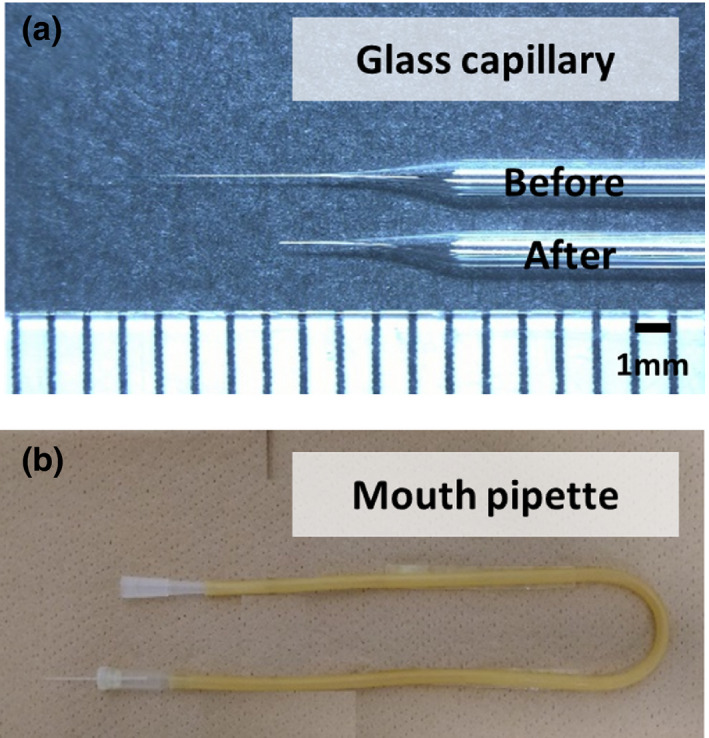
Preparation of the glass capillary and mouth pipette.(a) Cutting the tip of the capillary with scissors before injection. (b) Mouth pipette

We usually use a puller and glass capillaries to prepare micropipettes for microinjection into the oviduct. Conditions for preparing the injection capillary are as follows: Puller, Narishige PN‐31; glass capillary, Drummond #1‐000‐0300, HEATER 80, MAGNET‐SUB 30, or MAGNET‐MAIN 60–80, depending on the season and humidity. Before injection, cut off the tip of the capillary with scissors (Figure 3a). –Tip 1.

#### 2.5.2 Mouth pipette (Figure 3b)

Many types of mouth‐pipetting devices can be used. In our laboratory, we connect the aspirator tube assembly (for the calibrated micropipette) and the glass capillary. –Tip 2.

#### 2.5.3 Design of the specific guide RNA

Design the guide RNA using CHOPCHOP (Labun et al., 2019) (https://chopchop.cbu.uib.no/) and CRISPOR (Concordet & Haeussler, 2018) (http://crispor.tefor.net/). Choose the guide RNA by focusing on the “Efficiency” and “Off‐targets” parameters.

#### 2.5.4 CRISPR/Cas9 solution

1. Prepare 200 µM crRNA and tracrRNA in the Duplex buffer (IDT).
2. Mix equal volumes of the crRNA and tracrRNA, and heat the mixture at 95°C for 5 min.
3. Place the mixture at room temperature (20–25°C) for more than an hour before electroporation. –Tips 3, 4

#### 2.5.5 Tetramethylrhodamine‐labeled dextran (for GONAD practice)

Prepare 0.5 mg/ml tetramethylrhodamine‐labeled dextran and 0.1% trypan blue diluted in Opti‐MEM. –Tip 5

#### 2.5.6 Anesthetic agent

Mix 2 ml of midazolam (5.0 mg/ml), 2.5 ml of Vetorphale (5.0 mg/ml), 0.75 ml of Domitor (1.0 mg/ml), and 19.75 ml of 0.6% NaCl solution. –Tip 6

## 3 PROCEDURE (Figure 1)

### 3.1 Preparation of mice or rats (day −1, pm ~day 0, am)

1. Mate pre‐estrous females with males, without super‐ovulation. Choose females showing signs of pre‐estrus by examining the color, moistness, and degree of swelling of the vagina (Behringer, 2014; Champlin et al., 1973). –Tip 7
2. On the following morning, confirm the presence of copulation plugs by visual inspection and use these females for electroporation experiments.

### 3.2 Preparation of the CRISPR/Cas9 mixture (15 min before GONAD)

**FIGURE 4:**
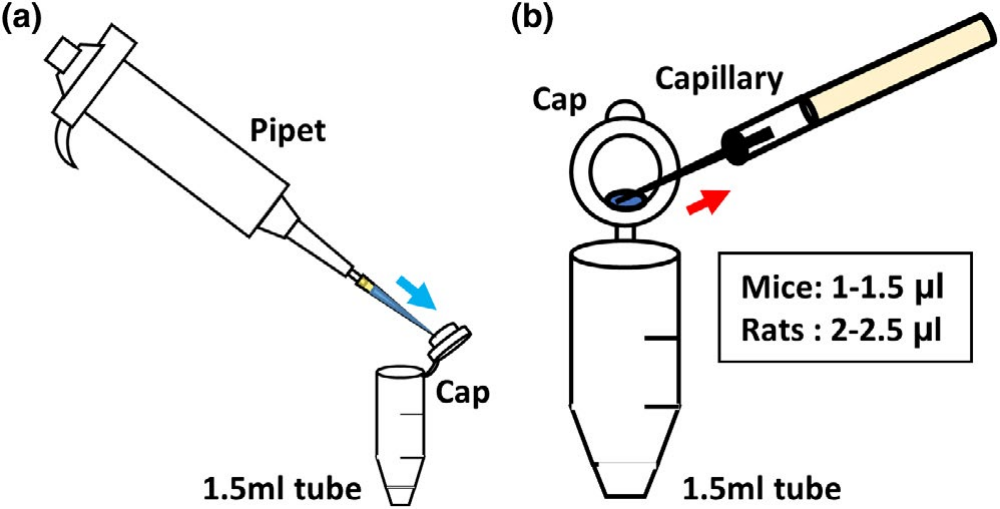
Preparation of the CRISPR/Cas9 mixture. (a) Mixing a solution on the inner wall of a cap. (b) Loading the solution into a capillary from the cap. Note: 1–1.5 µl (mouse) and 2–2.5 µl (rat) of the electroporation solution was injected into the oviductal lumen

1. Mix the components to a final concentration of 30 µM (for crRNA/tracrRNA), 1 mg/ml (for Cas9 protein), and 1 µg/µl (for ssODN) diluted in Opti‐MEM (Figure 4). –Tip 8.
2. Incubate for 5 min at room temperature. –Tip 4.

### 3.3 GONAD (day 0, pm) –Tip 9

#### 3.3.1 Surgical procedure (Movie S1)

1. Measure the body weight of the females.
2. Inject an anesthetic agent intraperitoneally (mice; 0.1 ml/10 g, rats; 0.5 ml/100 g).
3. Confirm anesthesia based on whether the mice or rats respond.
4. Spray rubbing alcohol on the dorsal skin.
5. Make a dorsal incision at the central portion of the back skin.
6. Make an incision in the muscle layer on the left side.
7. Place the exposed ovary, oviduct, and part of the uterus on the back.
8. Move the female under a dissecting microscope.

#### 3.3.2 Injection (Figure 5b, Movie S2)

**FIGURE 5:**
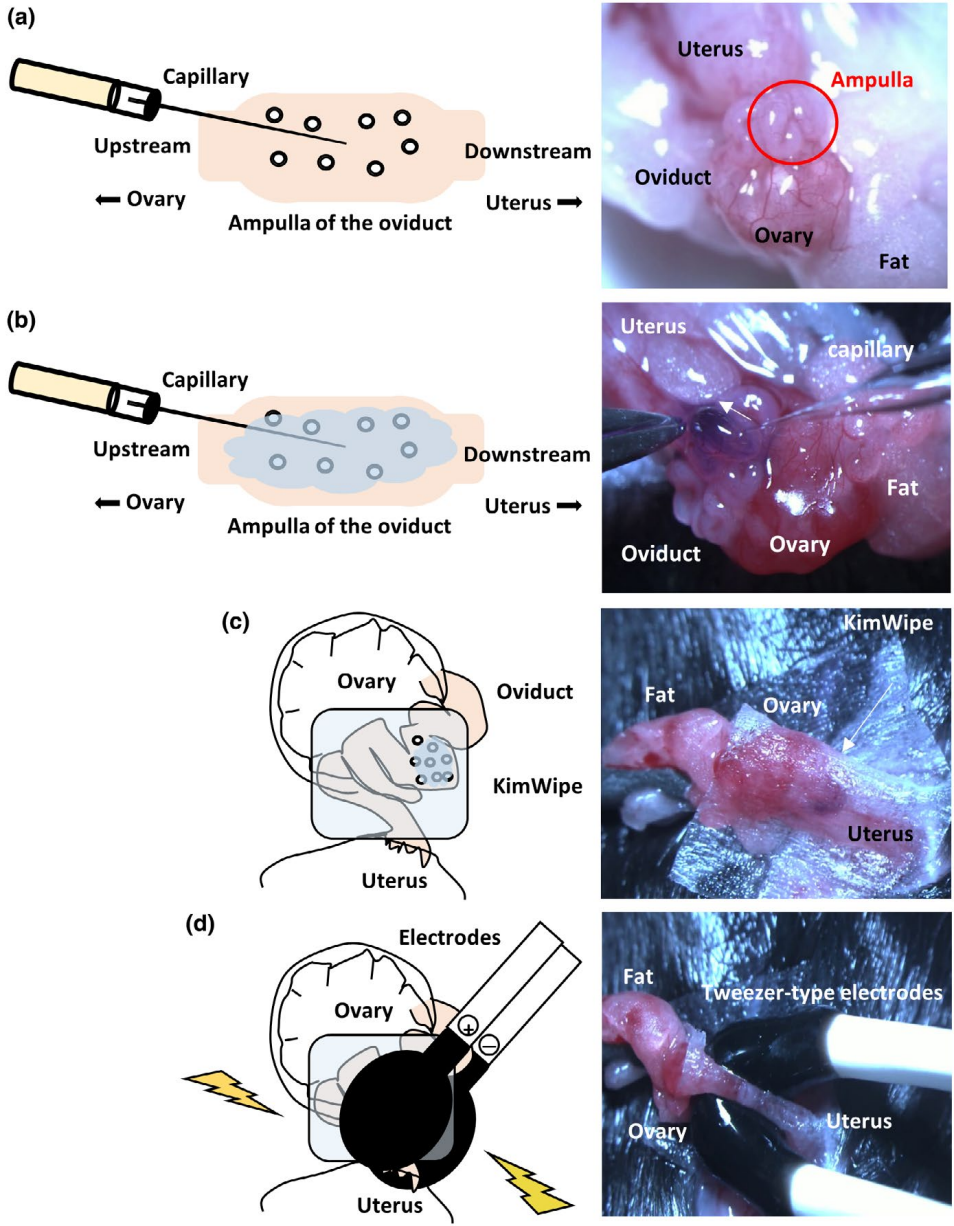
Injection and electroporation procedures. (a, b) Injection of the solution into the oviduct lumen upstream of the ampulla. (c) Covering the oviduct with a piece of a wet KimWipe soaked in PBS. (d) Applying pulses, after holding the oviduct between two electrodes

9 Fix the position of the ovary and oviduct to anchor the adipose tissue with an aorta clamp while observing under a microscope (Figure 5a).
10 Find the ampulla of the oviduct (to position the swollen oviduct), set the ampulla in the center, and confirm the upstream and downstream regions of the ampulla (Figure 5a,b). –Tip 10
11 Load the CRISPR/Cas9 mixture (mice, 1–1.5 µl; rats, 2–2.5 µl) into the injection capillary (Figure 4b).
12 Inject the solution into the oviductal lumen upstream of the ampulla with a mouth pipette (Figure 5b).

#### 3.3.3 Electroporation (Figure 5c,d, Movie S3)

**FIGURE 6:**
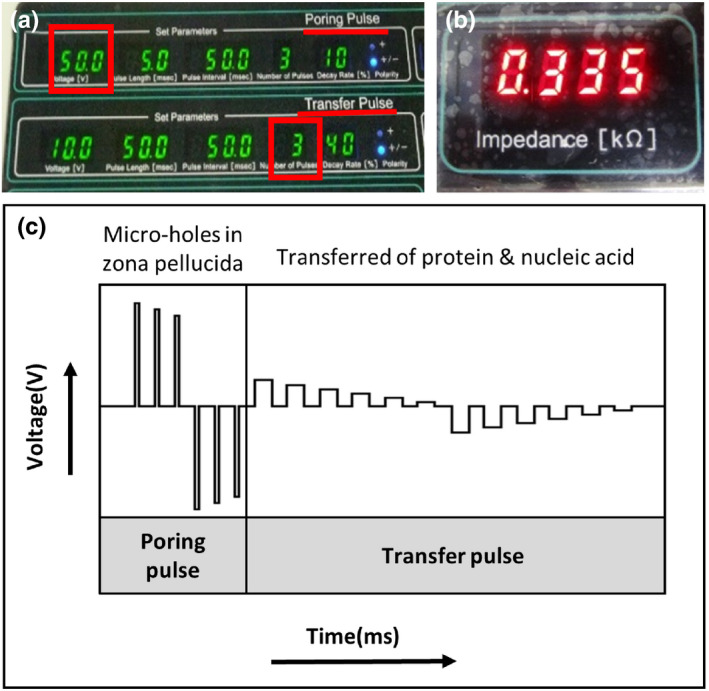
Electroporation conditions of the NEPA21 electroporator. (a–c) Electroporation conditions. (a) The electroporation parameters used were as follows: poring pulse, 50, 40, or 30 V (* in red square), 5 ms pulse, 50 ms pulse interval, three pulses, 10% decay (± pulse orientation); transfer pulse, 10 V, 50 ms pulse, 50 ms pulse interval, three or six pulses (# in red square), 40% decay (± pulse orientation). Note: Voltage with poring pulse: 30–50 V, Number of pulses with transfer pulse: 3 (mice) or 6 (rats). (b) Checking impedance before applying square pulses; optimal value, 0.15–0.50 kΩ. (c) Electroporation pulses by NEPA21

Electroporation conditions: see Figure 6a

Key parameters: –Tip 11; also see Anticipated Results.

Poring pulse: Voltage, 30–50 V.

Transfer pulse: Number of pulses, 3 (mice) or 6 (rats).

13 Cover the oviduct with a piece of a wet KimWipe towel soaked in PBS (Figure 5c).
14 Dip the tweezer‐type electrodes in PBS.
15 Hold the oviduct between the electrodes (Figure 5d).
16 Check the “Impedance” before applying square pulses (Figure 6b). –Tip 12
17 Apply square pulses using the electroporator. –Tip 13
18 Remove the KimWipe and aorta clamp.
19 Return the tissues to their original position.
20 Repeat steps 6–19 on the other side of the oviduct.
21 Close the skin using a suture wound clip.
22 Maintain the mice/rats in a cage at 37°C until they are awake.

### 3.4 Post‐GONAD experiment

#### 3.4.1 For GONAD practice (tetramethylrhodamine‐labeled dextran) (day 1–2)

Isolate the embryos from the treated females, and examine presence or absence of fluorescence 1 day (2‐cell embryos) or 2 days (~4‐cell embryos) after in vivo electroporation. –Tip 14.

**FIGURE 7:**
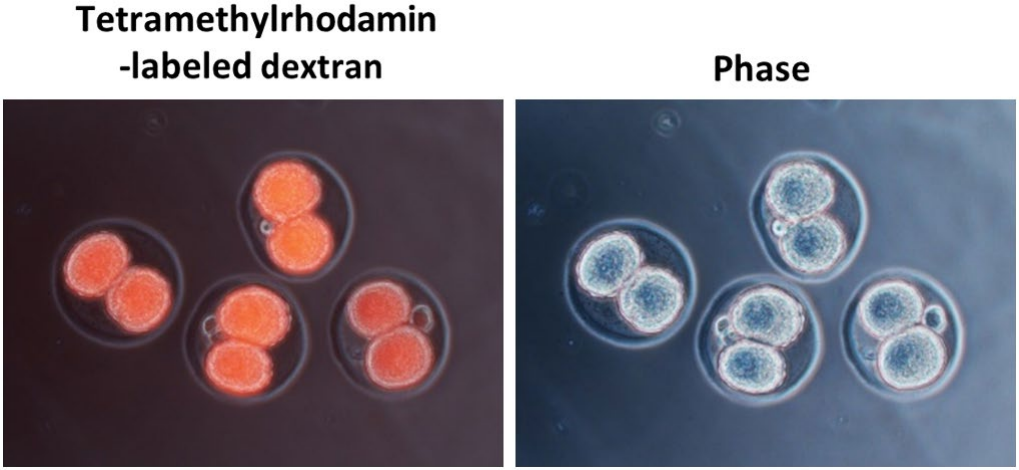
Fluorescence analysis with tetramethylrhodamine‐labeled dextran

1. Euthanize the pregnant mice or rats at 1 or 2 days after in vivo electroporation, and collect the organs connecting the ovary, oviducts, and upper portion of the uterus.
2. Wash embryos with PBS, and place them in a 60‐mm dish.
3. Insert a 30 G needle attached to a 1‐ml syringe containing 10% FBS/PBS into the oviductal lumen.
4. Flush out 10% FBS/PBS while under microscopic observation.
5. Collect the embryos with a pipette.
6. Observe the embryos with a fluorescence inverted microscope (Figure 7).

#### 3.4.2 For genome‐edited mice and rats (CRISPR/Cas9) (days 35–48)

House the treated females and offspring, and analyze the genotype. –Tip 15.

## 4 TIPS

**FIGURE 8:**
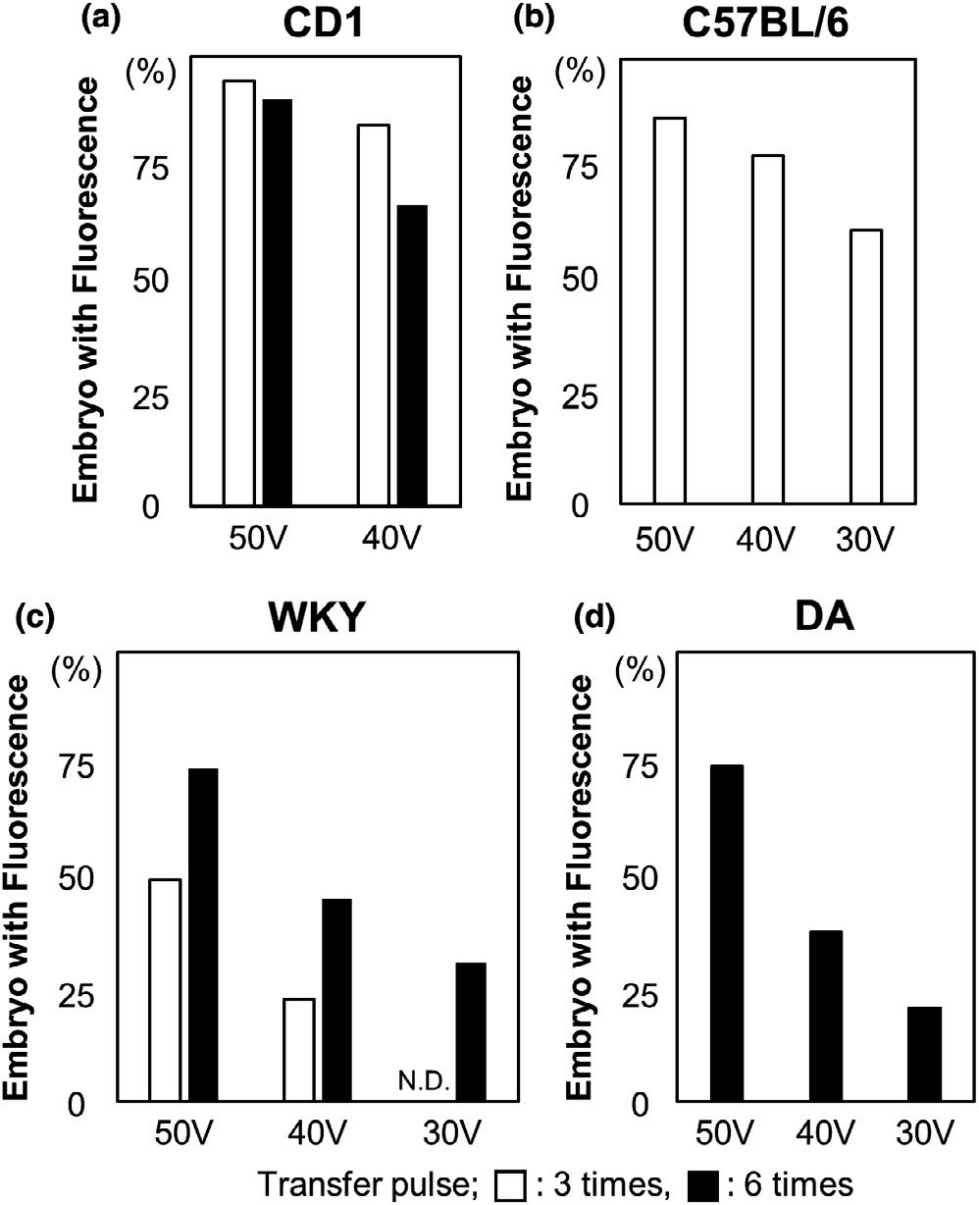
Determination of optimal electroporation efficiency. (a–d) Electroporation efficiency in (a) CD1 mice, (b) C57BL/6 mice, (c) WKY rats, and (d) DA rats. (c, d) are reproduced from (Kobayashi et al., 2018) under a Creative Commons Attribution License (CC BY 4.0)

1. The glass capillary tube used for GONAD does not need to be of high quality, unlike those required for embryo microinjections.
2. This mouth pipette is also used for transferring embryos on 1 or 2 days after electroporation.
3. Point: Can be stored at −30°C until use.
4. Note: For the preparation of CRISPR/Cas9 regents, the Alt‐R CRISPR‐Cas9 system (IDT) was used in accordance with the manufacturer’s protocol.
5. Point: Mix the components before injection. Usually, this mixture can be stored at 4°C for approximately 1 week.
6. Point: Can be stored at room temperature for up to 2 weeks.
7. Caution! In our initial trial, we could hardly obtain pups from the super‐ovulated female mice (C57BL/6) and rats. Therefore, we used estrous female mice and rats without super‐ovulation.
8. Point: Mix CRISPR/Cas9 reagents in a 1.5‐ml tube on the inner wall of the “cap” (Figure 5). It is very easy to load this into the capillary when the solution is injected.
9. Important! In our laboratory, mice and rats were housed under a 12 h–12 h light–dark cycle (light on at 6:00 and off at 18:00). For the GONAD experiment, females were injected at E0.7 (mice, at around 15:00) or E0.75 (rats, at around 16:00). The GONAD experiments conducted with E0.4–0.5 (before 12:00) failed (Kobayashi et al., 2018; Ohtsuka et al., 2018). We strongly recommend that the experimental road map be refined depending on the laboratory.
10. Point: To distinguish regions upstream or downstream of the ampulla in the oviduct, find the infundibulum connecting the ovary. Trace the oviduct from the ovary to the uterus to find the ampulla. The ovary side is upstream of the ampulla, and the uterus side is downstream of the ampulla. See also Figure 5.
11. Important! Optimize the electroporation conditions depending on the strain of the mice, rats, or other mammals. We checked numerous different conditions of electroporation for mice and rats. For more details, see “Anticipated Results”.
12. Point: Optimal value, 0.15–0.50 kΩ. Aim to successfully hold the oviduct between the electrodes to measure the impedance.
13. Point: On successful delivery of the pulses, a slight reflex movement of the mouse or rat itself and air bubbles around the electrode due to Joule heating can be observed.
14. Note: After detecting fluorescence with more than 50% efficiency, advance to the next step (genome editing: CRISPR/Cas9).
15. Troubleshooting:
  No pups or a low number of pups:
    Apply higher current voltage. Optimize electroporation conditions for mice or rats. See also Figure 8.
    Use super‐ovulated females. Use estrous female mice or rats without super‐ovulation.
  Low genome‐editing efficiency:
    Failure of injection and/or electroporation. Practice GONAD with tetramethylrhodamine‐labeled dextran after detecting fluorescence with more than 50% efficiency.
    The chosen guide RNA is wrong. Change to the other guide RNA. See also section 3.5.3.
    Homozygous mutant mice or rats are lethal. Efficiency of genome editing by GONAD is often high, and therefore, both alleles may be modified in these mice or rats. Lower the current voltage to edit only one allele.

## 5 ANTICIPATED RESULTS

### 5.1 Electroporation conditions

We examined six different conditions for electroporation (poring pulse voltage: 50, 40, or 30 V; number of transfer pulses: 3 or 6; see Figure 6a–c) with tetramethylrhodamine‐labeled dextran injected into mice and rats. One or two days after in vivo electroporation, embryos were isolated from the treated females and the presence or absence of fluorescence was examined (Figure 7). As shown in Figure 8a, the efficiency in the CD1 mouse stain reached over 90% with a poring pulse of 50 V (transfer pulse: 3, 94.6%; 6, 90.5%). A poring pulse of 40 V was adequate to obtain high efficiency (transfer pulse: 3, 84.8%; 6, 66.9%). In mice, the same efficiency could be reached with three or six transfer pulses (Figure 8a). No significant difference was detected in the gene‐editing efficiency between the CD1 and C57BL/6 strains of female mice (50 V, 86.8%; 40 V, 78.3%; 30 V, 61.5%; Figure 8b). Nonetheless, as viability of the manipulated C57BL/6 mouse embryos is often lower (Behringer, 2014), a poring pulse of 40 or 30 V for the C57BL/6 strain can be attempted if a poring pulse of 50 V yields few pups.

Although in mice the efficiency did not appear to differ between three and six transfer pulses, in rats six transfer pulses were more efficient than three (Figure 8c,d), as described in our previous report (Kobayashi et al., 2018).

These data suggest that the most suitable conditions are as follows:

- Mouse: Poring pulse voltage, 30–50 V; number of transfer pulses: 3
- Rat: Poring pulse voltage, 40–50 V; number of transfer pulses: 6

### 5.2 Gene editing

**FIGURE 9:**
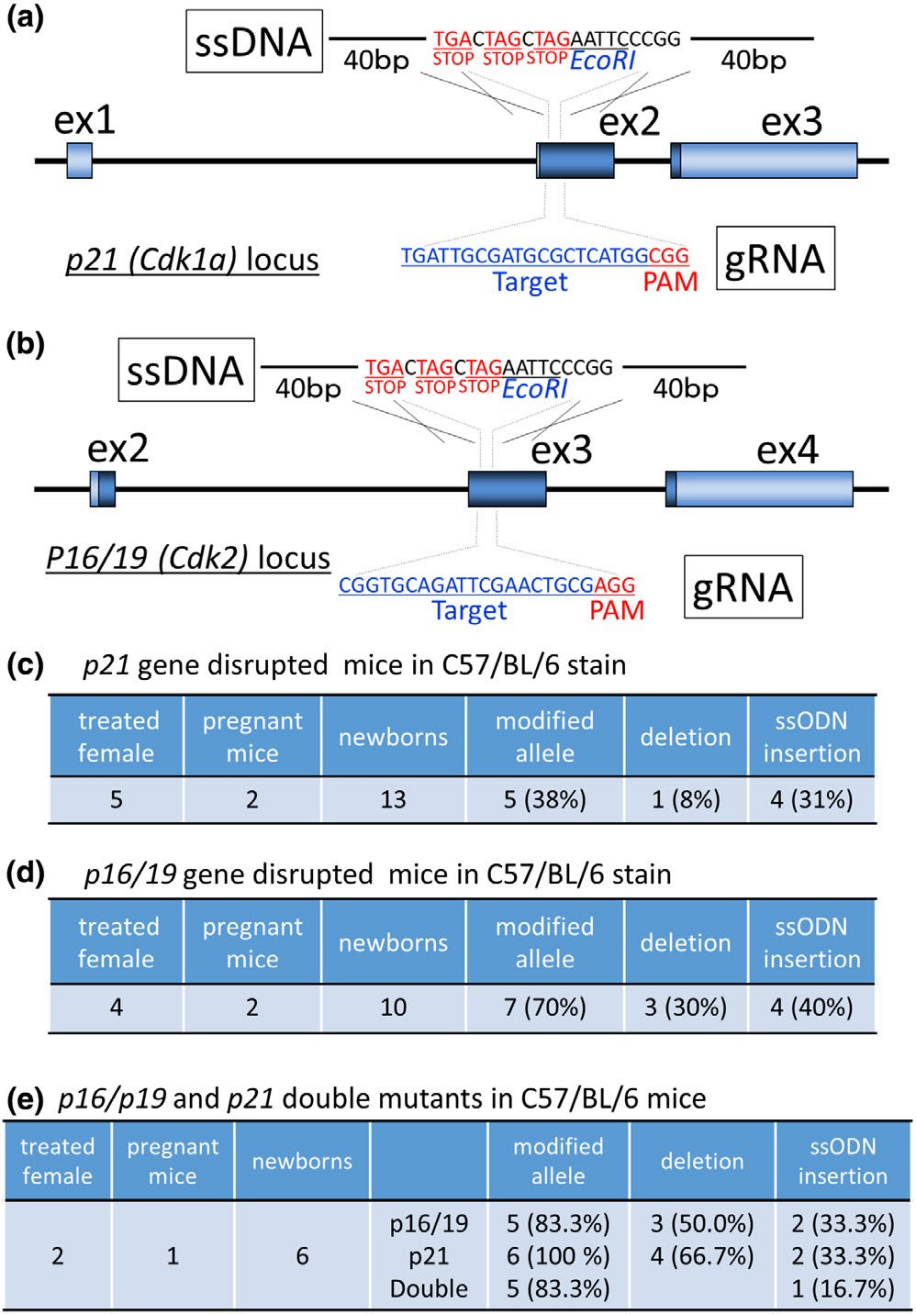
Production of *p21* and *p16/19* mutant mice of the C57BL/6 stain. (a, b) The targeting strategy to disrupt the (a) *p21* or (b) *p16/19* genes. The gRNA target sequence, PAM, and ssODN are shown. (c–e) Genome‐editing efficiency by *i*‐GONAD of the (c) *p21*, (d) *p16/19*, and (e) *p21; p16/19* double knock‐out mice. (a–d) are reproduced from (Koyano et al., 2019; Ohtsuka et al., 2018) under a Creative Commons Attribution License (CC BY 4.0)

In our laboratory, the Cas9 protein, guide RNA, and ssDNA of 20 bases including three tandem stop codons were injected for producing gene‐disrupted mice and rats via the knock‐in approach (Figure 9a,b) (Koyano et al., 2019; Namba et al., 2021). As shown in Figure 8c,d, we were able to obtain gene‐edited mice (*p21* gene, four pups, 31%; *p16/19* gene, four pups, 40%). We also obtained double‐mutant mice by using a mixture of two CRISPR/Cas9 reagents (Figure 9e). Fewer than five females were used to produce these knock‐in mice, suggesting that *i‐*GONAD is a useful method from the perspective of the 3R principles.

**FIGURE 10:**
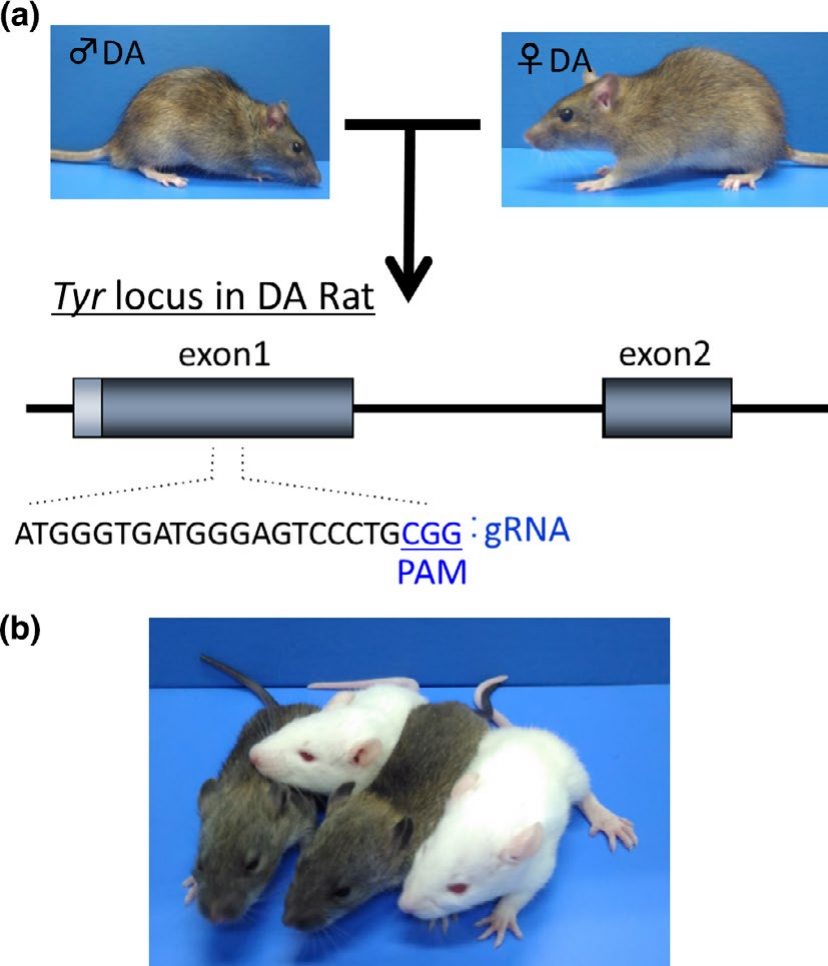
Production of *Tyr* knock‐out rats with biallelic disruption. (a) Genome editing for knock‐out rats. rGONAD was performed in 0.75‐day pregnant DA female rats crossed with DA male rats. The gRNA target sequence and PAM at the *Tyr* locus are shown. (b) Some of the genome‐edited rats had albino coats

The rGONAD method is also highly efficient in both knock‐out and knock‐in trials, as shown in our previous report (Kobayashi et al., 2018), and we succeeded in generating a novel Alport syndrome rat model with this method (Namba et al., 2021). In addition, many pups obtained by rGONAD had mutations in both alleles (Figure 10, Table 1), suggesting that this method allows efficient production of rats with biallelic disruptions, which is highly useful for the study of gene functions using F0 pups.

**TABLE 1:** *Tyr*‐mediated mutations in DA rats

Strain	Poring pulse voltage (V)	No. injected	No. pregnant	Pups (A)	Het (B) (%: B/A)	Homo (C) (%: C/A)
DA × DA	50	17	12	70	4 (5.7)	16 (22.9)
	40	15	8	42	0	7 (16.7)

Abbreviations: Het, heterozygous mutant; Homo, homozygous mutant.

## Supporting information

Movie S1

Movie S2

Movie S3

Supplementary Material
