# Supplementary material for: GONAD: A new method for germline genome editing in mice and rats
Source: Dev Growth Differ. 2021 Oct 5;63(8):439–47. doi: 10.1111/dgd.12746 (PMC11520964; doi:10.1111/dgd.12746)
Supplement: Supplementary file 4 — Supplementary Material [file DGD-63-439-s004.docx]

**Movie S1.** Surgical steps taken to expose the ovary, oviduct, and proximal region of the uterus.

**Movie S2.** Instillation of the solution containing tetramethylrhodamine-labeled dextran.

**Movie S3.** Electroporation with a NEPA21 electroporator.
